# Ecoepidemiology of Cutaneous Leishmaniasis Outbreak, Israel 

**DOI:** 10.3201/eid1409.071100

**Published:** 2008-09

**Authors:** Shepherd Roee Singer, Nitsa Abramson, Hanna Shoob, Ora Zaken, Gary Zentner, Chen Stein-Zamir

**Affiliations:** Jerusalem District Health Office, Jerusalem, Israel

**Keywords:** Climate, cutaneous leishmaniasis, global warming, Leishmania tropica, outbreak, dispatch

## Abstract

A total of 161 cases of cutaneous leishmaniasis caused by *Leishmania tropica* occurred in the Jerusalem district during 2004–2005; 127 (79%) cases were in a town just outside Jerusalem. Environmental models suggest that in the context of global warming, this outbreak has the potential to extend into Jerusalem.

Leishmaniasis is a zoonotic infection in which parasites of the genus *Leishmania* are transmitted from rodents and small mammals to *Phlebotomus* species sandfly vectors. Humans may inadvertently enter the zoonotic cycle and contract cutaneous leishmaniasis (CL). This disease manifests as a chronic ulcer, potentially leaving unattractive scars.

CL incidence throughout the 1990s remained at a relatively stable 0.5–2.5 cases/100,000 (*1*). *Leishmania major* is found in low-lying arid and semiarid deserts and has been responsible for most cases in Israel (*2*). *L*. *tropica,* typically anthroponotic, is more common in suburbs and villages, although in hilly rural areas, mammals may act as reservoirs (*3*). The sandfly *Phlebotomus papatasi* is vectorially competent for *L*. *major* only (*4*), and *Ph*. *sergenti* is specific for *L*. *tropica* (*5*). Sandflies are usually found within 200 m of their source.

Since the 1990s, *L*. *tropica*, either alone or in conjunction with *L*. *major*, has been implicated in several outbreaks of CL in the western regions of the Jordan Valley (*6*). We report a large outbreak of CL caused by *L*. *tropica* in a town on the outskirts of Jerusalem.

## The Study

Jerusalem is located atop the Judean Hills, on the edge of the Judean Desert. The desert drops off steeply to the east, falling 1,200 m over a course of 20 km to a nadir of –400 m on the shores of the Dead Sea, the lowest point on land on Earth. On the edge of this region is Ma’ale Adumim, 5 km east of Jerusalem (population ≈33,000). The town is built along narrow ridges that fall away to deep ravines inhabited by wildlife. Houses on the periphery are often just meters from desert crags and crevices; none is more than 500 m from the wilderness.

All cases of leishmaniasis in Israel are required to be reported to the district health office, and a weekly national report is published by the Department of Epidemiology, Israeli Ministry of Health (*7*). We confirmed suspected cases by using stained smears, culture, or serologic analysis. Cases were plotted on the Ministry of Health geographic information system (Environmental Systems Research Institute, Redlands, CA, USA), adapted specifically for the Informatics and Computation Division of the Ministry of Health (Systematics Technologies Ltd., Tel Aviv, Israel). National population data were derived from the Israeli Central Bureau of Statistics. Local population data were supplied by the town municipality, and neighborhood incidence rates were derived and averaged for 2004–2005. Patients were interviewed by using a standardized national CL questionnaire (Technical Appendix). Simple rate ratios (RRs) were calculated where applicable.

A total of 161 cases of CL were reported in the Jerusalem district in 2004 (n = 71) and 2005 (n = 90) compared with 1 or 2 cases in each previous year. Of the cases reported in 2005, microscopic examination was positive for 74 (82%). Forty-eight (53%) had positive cultures; 20 (41.6%) of these were serologically positive for *L*. *tropica*, and none was positive for *L*. *major*.

**Figure 1:**
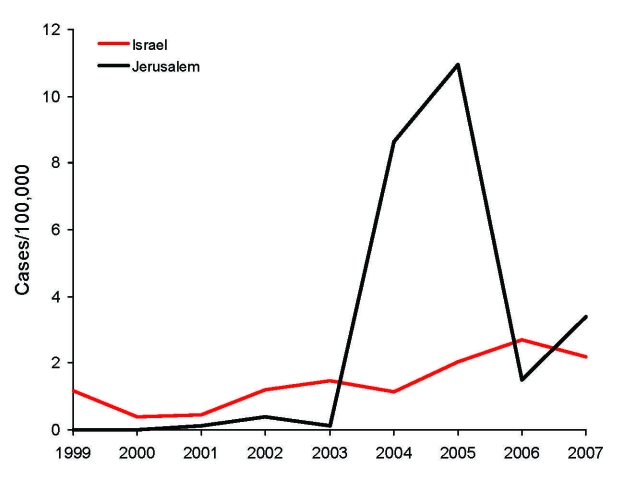
Incidence rates of cutaneous leishmaniasis for the Jerusalem district and Israel, 1999–2007. Rates for Israel do not include cases reported in the Jerusalem district.

Average annual incidence of CL in Israel (excluding the Jerusalem district) increased from 0.95/100,000 in 1999–2003 to 1.61/100,000 in 2004–2005 (RR 1.63). Over the same period, however, rates for the Jerusalem district increased from 0.13 to 9.7/100,000 (RR 74.7). Rates for the Jerusalem district were lower than those for the rest of Israel during 1999–2003 (RR 0.14) but substantially higher during 2004–2005 (RR 6.1). In 2006, rates for the Jerusalem district decreased, and national incidence continued to increase; 2007 showed a trend to an increased incidence in the Jerusalem district (Figure 1).

**Figure 2:**
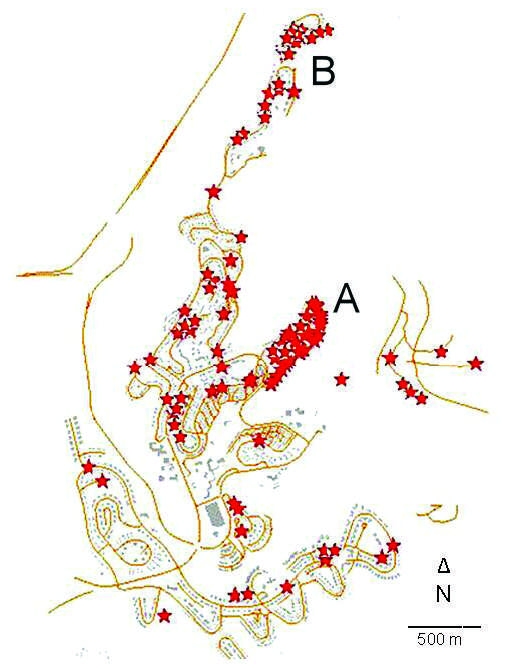
Geographic distribution of cutaneous leishmaniasis cases in Ma'ale Adumim, Israel, 2004–2005. Each star represents 1 case. In dense areas, some marks may be missing because of technical limitations. Wilderness ravines (white areas) reach within meters of houses at the periphery of the neighborhood in all directions. A and B indicate neighborhoods with the highest incidence rates.

Of the case-patients in the Jerusalem district, 54 (76%) in 2004 and 73 (81%) in 2005 occurred in Ma’ale Adumim, where the incidence was 214/100,000 in 2004–2005, compared with an annual average of 2/100,000 in preceding years. However, cases were not distributed evenly. The epicenter of the outbreak was a small neighborhood of 1,040 residents (A in Figure 2) in which 52 cases occurred over the 2-year period (attack rate 50/1,000); this attack rate was greater than in any other neighborhood. The second most affected neighborhood had 24 cases among 2,251 residents (attack rate 10.7/1000 for the 2-year period). All but 6 case-patients lived within 200 m of the ravines that encompass the town, and 3 of these case-patients had occupational exposure. In 2006 and 2007, 13 and 29 cases, respectively, were reported in the Jerusalem district; 5 (38%) and 9 (31%) of these case-patients, respectively, were infected in Ma’ale Adumim. The remaining cases were widely dispersed.

Age distribution of patients was not different from that of uninfected persons in the town (Table). More than one third of the patients (37.5%) reported a family member who was infected with CL in the same period. Most patients (62.2%) had >1 lesion, 70% reported having a private garden, 57.1% lived adjacent to public parks, 52.7% had a home that faced a wilderness area, 45.8% reported a construction site near their home, and 65.1% had intact insect screens on their windows. Eight patients (14%) (6 in neighborhood A and 2 in neighborhood B) reported seeing hyraxes near their homes (Figure 2).

**Table:** Age distribution of cutaneous leishmaniasis case-patients in the Jerusalem district, Israel, 2004–2005

Age group, y	No. (%)
<1	4 (2.5)
1–4	12 (7.5)
5–14	40 (24.8)
15–44	46 (28.6)
45–64	29 (18.0)
>65	19 (11.8)
Unknown	11 (6.8)
Total	161 (100)

## Conclusions

We report a large outbreak of CL in Israel caused by *L*. *tropica* that was centered on a town just outside Jerusalem. This outbreak was observed in the context of increasing rates of CL in Israel. During the outbreak, highly visible environmental intervention and active surveillance were undertaken, which may have introduced detection bias, but it is unlikely that these alone could explain the dramatic increase in incidence.

The association between CL outbreaks and urban development has been noted repeatedly, and construction waste and soil humidity are considered intermediaries (*8**,**9*). A study in Colombia found that habitat degradation negatively affected phlebotomine populations but that medically important sandfly species were able to exploit modified environments (*10*). Ma’ale Adumim has undergone rapid development and expansion over the past decade, and sandflies are abundant in the area. A 2005 study collected 80,000 sandflies near the town, of which 85% were *Ph*. *sergenti* (*11*), the vector for *L*. *tropica*. Hyraxes were sighted most frequently in the worst CL-affected neighborhoods. Although environmental investigation is ongoing, we suspect that hyraxes infesting building sites were the source of this outbreak. This hypothesis is in accordance with current knowledge that associates *L*. *tropica* with urban outbreaks and hilly terrain. However, the pattern of this outbreak supports a zoonotic rather than anthroponotic source.

In 2006, Chaves and Pascual reported that climate was a valuable covariate in predicting incidence of CL (*12*). A 1996 computerized model examined the effect of warming of 1°C, 3°C, and 5°C on the likelihood of CL transmission at 115 southwest Asian sites. Sandflies are not known to reproduce in Jerusalem, but Cross and Hyams suggest that Jerusalem could support endemic transmission if the average temperature increased by 1°C (*13*). Since that model was proposed, the average temperature in Jerusalem has increased by ≈1°C (*14*). The spate of recent outbreaks suggests that *L*. *tropica* is no longer an emerging pathogen but rather that it is an established pathogen in Israel. The proximity of the outbreak to Jerusalem, in light of the trend toward global warming (*15*), makes an outbreak of CL in Jerusalem a real and disturbing prospect.

## Supplementary Material

Technical Appendix Cutaneous Leishmaniasis questionnaire
