# Supplementary material for: Ecoepidemiology of Cutaneous Leishmaniasis Outbreak, Israel
Source: Emerg Infect Dis. 2008 Sep;14(9):1424–6. doi: 10.3201/eid1409.071100 (PMC2603121; doi:10.3201/eid1409.071100)
Supplement: Technical Appendix — Cutaneous Leishmaniasis questionnaire [file 07-1100_Techapp-s1.pdf]

All rights reserved Ministry of Health, Israel and Ministry Environmental Protection, Israel

Cutaneous Leishmaniasis questionnaire

1. District Health Office ________________
2. Date of notification to District Health Office ____/____/____
3. Name of reporting physician ____________________
4. Place of employment of reporting physician ___________________

Patient personal details

Surname _____________ Given name(s)__________ I.D. ________________

D.O.B. __/__/____ Sex [1] Male [2] Female

Nationality: [1] Jew [2] Non-Jew [3] Unknown Health insurance provider _________

Occupation (children – record daytime placement): ___________________________

Current address: City/village/settlement ___________________________________

Street _________________________ House no.___

Clinical and laboratory information

Date of appearance of first signs of the disease: __/__/____

Date of definitive diagnosis: __/__/____

Site of lesion	Nature of lesion	No. lesions ___
Face [1] Yes [2] No	Ulcer [1] Yes [2] No	
Limbs [1] Yes [2] No	Nodule [1] Yes [2] No	
Other [1] Yes [2] No	Other [1] Yes [2] No	
If yes, specify __________________	If yes, specify ____________	

All rights reserved Ministry of Health, Israel and Ministry Environmental Protection, Israel

Was a laboratory diagnosis made? [1] Yes [2] No [3] Do not know

If so, what were the results of the following tests? Please record as follows:

[1] Positive result [2] Negative result [3] Not carried out

Test	Result	Date of test
1. Direct smear	[ ]	__/__/____
2. Histologic examination	[ ]	__/__/____
3. Culture	[ ]	__/__/____
4. Serology	[ ]	__/__/____
5. *Leishmania* skin test	[ ]	__/__/____
6. PCR	[ ]	__/__/____

Type of *Leishmania* (if identified):

1. *tropica*
2. *major*
3. *infantum*
4. Other ______________

1. Epidemiologic data

Have you moved house in the 12 months preceding the diagnosis of the illness?

[1] Yes [2] No [3] Don't know

If you have:

Previous address ____

Date of move ____________________

Sites/areas of presumed infection* (specify precise address and period spent in that place)

All rights reserved Ministry of Health, Israel and Ministry Environmental Protection, Israel

Address   Period spent at that place

____

____

____

* e.g., Near home, on military reserve duty, holidays, sleeping out in the open, sitting on porch or in garden

Were you bitten by an insect other than a mosquito? [1] Yes [2] No [3] Don't know

If yes, describe the insect ____

If yes, please fill out the following table:

Place (precise location and description, e.g., garden, lawn, beach etc.)	Season when bitten	How do you know you were bitten?	Time of day when bitten	Were other people in the same place bitten at the same time?
	☐ Spring<br>☐ Summer<br>☐ Fall (Autumn)<br>☐ Unknown	☐ Pricking feeling<br>☐ Itch<br>☐ Mark on skin<br>☐ Other	☐ Evening<br>☐ Night<br>☐ Morning<br>☐ Unknown	☐ No<br>☐ Only a few<br>☐ Most of them<br>☐ Unknown
	☐ Spring<br>☐ Summer<br>☐ Fall (Autumn)<br>☐ Unknown	☐ Pricking feeling<br>☐ Itch<br>☐ Mark on skin<br>☐ Other	☐ Evening<br>☐ Night<br>☐ Morning<br>☐ Unknown	☐ No<br>☐ Only a few<br>☐ Most of them<br>☐ Unknown

All rights reserved Ministry of Health, Israel and Ministry Environmental Protection, Israel

	☐ Spring<br>☐ Summer<br>☐ Fall (Autumn)<br>☐ Unknown	☐ Pricking feeling<br>☐ Itch<br>☐ Mark on skin<br>☐ Other	☐ Evening<br>☐ Night<br>☐ Morning<br>☐ Unknown	☐ No<br>☐ Only a few<br>☐ Most of them<br>☐ Unknown

Do you use personal protective measures against mosquito, sandfly, or other insect bites?

Fans	Insect repellent on skin (in the home)	Insect repellent on skin (outside the home)	Vaporizing tablets or liquid	Repellent candles or coils	Spraying within the house	Spraying outside the house	Other
☐ Yes<br>☐ No<br>☐ Sometimes	☐ Yes<br>☐ No<br>☐ Sometimes	☐ Yes<br>☐ No<br>☐ Sometimes	☐ Yes<br>☐ No<br>☐ Sometimes	☐ Yes<br>☐ No<br>☐ Sometimes	☐ Yes<br>☐ No<br>☐ Sometimes	☐ Yes<br>☐ No<br>☐ Sometimes	☐ Yes<br>☐ No<br>☐ Sometimes

Are there screens on the windows and doors in your home? [1] Yes [2] Partial [3] No

If yes, are they intact and in good condition? [1] Yes [2] No

Does your home have a private garden? [1] Yes [2] No [3] Don’t know

Is there a public park near your home? [1] Yes [2] No [3] Don’t know

Do you tend to spend time outdoors: in the garden, near your home, in the neighborhood ? [1] Yes [2] No [3] Don’t know

All rights reserved Ministry of Health, Israel and Ministry Environmental Protection, Israel

During what hours of the day? [1] Morning [2] Afternoon [3] Evening [4] After sunset or at night

Information on other family members, relatives, community members, hiking companions etc. who visited or were together with the infected person in the above places, and developed cutaneous leishmaniasis

Name	Sex	Age	Relationship	Address	Date disease onset

Do you consent to the information obtained from this interview being given to inspectors from the Environmental Protection Department?

Yes/No

If yes, please sign here ______________________________

If the consent was by telephone, please state.

Name of interviewer __________________________________

Position ____
